# Characteristics and outcomes of patients who did not respond to a national spine surgery registry

**DOI:** 10.1186/s12891-023-06267-3

**Published:** 2023-03-04

**Authors:** Simran Kaur, Ole Kristian Alhaug, Filip C. Dolatowski, Tore K. Solberg, Greger Lønne

**Affiliations:** 1 Department of Orthopedic Surgery, Martina Hansens Hospital, Sandvika, Norway; 2 Innlandet Hospital Trust, Brumunddal, Norway; 3 Akershus University Hospital, Nordbyhagen, Norway; 4 Norwegian University of Science and Technology, Trondheim, Norway; 5 Division of Orthopedic Surgery, Oslo University Hospital, Oslo, Norway; 6 Department of Neurosurgery & Norwegian Registry for Spine surgery, University Hospital of North Norway, Tromsø, Norway; 7 Department of Clinical Medicine, The Arctic University of Norway (UiT), Tromsø, Norway

**Keywords:** Loss to follow-up, Spine surgery, Attrition bias, Registry, Non-response, Lumbar spinal stenosis

## Abstract

**Background:**

Loss to follow-up may bias outcome assessments in medical registries. This cohort study aimed to analyze and compare patients who failed to respond with those that responded to the Norwegian Registry for Spine Surgery (NORspine).

**Methods:**

We analyzed a cohort of 474 consecutive patients operated for lumbar spinal stenosis at four public hospitals in Norway during a two-year period. These patients reported sociodemographic data, preoperative symptoms, and Oswestry Disability Index (ODI), numerical rating scales (NRS) for back and leg pain to NORspine at baseline and 12 months postoperatively. We contacted all patients who did not respond to NORspine after 12 months. Those who responded were termed responsive non-respondents and compared to 12 months respondents.

**Results:**

One hundred forty (30%) did not respond to NORspine 12 months after surgery and 123 were available for additional follow-up. Sixty-four of the 123 non-respondents (52%) responded to a cross-sectional survey done at a median of 50 (36–64) months after surgery. At baseline, non-respondents were younger 63 (SD 11.7) vs. 68 (SD 9.9) years (mean difference (95% CI) 4.7 years (2.6 to 6.7); *p* =  < 0.001) and more frequently smokers 41 (30%) vs. 70 (21%) RR (95%CI) = 1.40 (1.01 to 1.95); *p* = 0.044. There were no other relevant differences in other sociodemographic variables or preoperative symptoms. We found no differences in the effect of surgery on non-respondents vs. respondents (ODI (SD) = 28.2 (19.9) vs. 25.2 (18.9), MD (95%CI) = 3.0 ( -2.1 to 8.1); *p* = 0.250).

**Conclusion:**

We found that 30% of patients did not respond to NORspine at 12 months after spine surgery. Non-respondents were somewhat younger and smoked more frequently than respondents; however, there were no differences in patient-reported outcome measures. Our findings suggest that attrition bias in NORspine was random and due to non-modifiable factors.

**Supplementary Information:**

The online version contains supplementary material available at 10.1186/s12891-023-06267-3.

## Background

Medical registries provide clinicians with large data sets of high external validity and complement randomized controlled trials that examine more targeted populations and treatments [1, 2]. Medical registries can guide decision-making and improve the quality of care by monitoring patient-reported outcome measures (PROMs) stratified by different populations, diagnoses, and treatments [3, 4]. Medical registries face higher attrition rates compared to clinical trials—rigorous attempts to attain data are costly and impractical in a registry setting [5]. Still, sufficient follow-up rates are crucial for the quality of registries, and awareness of follow-up rates is important when interpreting register data.

Non-respondents may systematically differ from respondents and introduce attrition bias that compromises the validity of register data [1, 6–8]. However, some studies suggest that non-response occurs at random [2, 4, 5]. The last assessment of non-respondents in NORspine was conducted in 2007 and reported a loss to follow-up of 22% at two years postoperatively and did not reveal any differences in outcomes between non-respondents and respondents [5]. This study was conducted before NORspine expanded to a national registry, and a reassessment is warranted. In order to assess the impact of attrition on NORspine data, we aimed to assess baseline characteristics and clinical outcomes for patients who responded at 12 months after surgery compared to those who did not.

## Methods

This cohort study was based on retrospective analyses of prospectively collected NORspine data. We compared baseline variables for patients who did not respond to NORspine at 12 months after surgery with those who had responded. We reached out to those who did not respond to NORspine at 12 months after surgery and performed an additional cross-sectional survey at a median of 50 (36–64) months after surgery. We assessed clinical outcomes for those who finally responded to our additional questionnaire. As an additional analysis, we also compared the baseline variables of the subgroup that never responded compared to those who responded to the additional cross-sectional survey.

### NORspine

All Norwegian hospitals that offer spine surgery are obliged to report to NORspine. Currently, 70% of all degenerative spine surgeries done in Norway are registered in NORspine [9]. NORspine is a consent-based register. Patients with primary infections of the spine, fractures of the spine, and patients who are unable to comprehend questionaries in Norwegian, are not invited to participate.

A NORspine dataset consists of both patient- and surgeon-reported variables. Patients complete a standardized questionnaire preoperatively on sociodemographic data such as age, sex, native language, level of education, and marital status. Patients also report preoperative symptoms, as assessed by validated PROMs: Oswestry Disability Index (ODI) ranging from 0 (minimal disability) to 100 (bedbound), Numeric Rating Scales (NRS) ranging from 0 (no pain) to 10 (worst imaginable pain) for back and leg pain, and quality of life as assessed by EuroQol 5 Dimension 3 level—0.59 (worse than dead) to 1,0 (perfect health) [10–13].

Surgeons report directly after the surgery on diagnoses, relevant comorbidities, and perioperative details such as the type of surgery. The NORspine sends follow-up questionnaires to patients at 3 and 12 months after surgery by regular mail, including one reminder if the patient does not reply. Patients report directly to NORspine at follow-ups using PROMs (ODI, NRS back and leg pain, EQ5D, and Global Perceived Effect (GPE)—a seven-point Likert scale (1 = completely recovered, 2 = much improved, 3 = slightly improved, 4 = unchanged, 5 = slightly worse, 6 = much worse, 7 = worse than ever) [14].

### Data collection

We analyzed prospectively collected NORspine data on patients operated for lumbar spinal stenosis (LSS) at four hospitals between January 1^st^, 2015 and December 31^st^, 2016. Patients who consented to participate in NORspine completed questionnaires at baseline. The NORspine registry then mailed similar questionnaires to patients at 12 months postoperatively. Patients responded directly to NORspine without the involvement of the treating center. NORspine routinely sends one postal reminder to those who do not respond before they are considered non-respondents. We engaged the NORspine office to reach out to those who did not respond at 12 months after surgery. The 12 months postoperative questionnaire was sent once again. We also sent one reminder by mail and one by SMS to those who still did not respond. Patients that responded at 12 months postoperatively are termed respondents, while those who did not respond are termed non-respondents. Those who finally responded are termed “responsive non-respondents”, and those who never responded to any contact are termed “resistant non-respondents”.

### Baseline and outcome measures

**Fig. 1:**
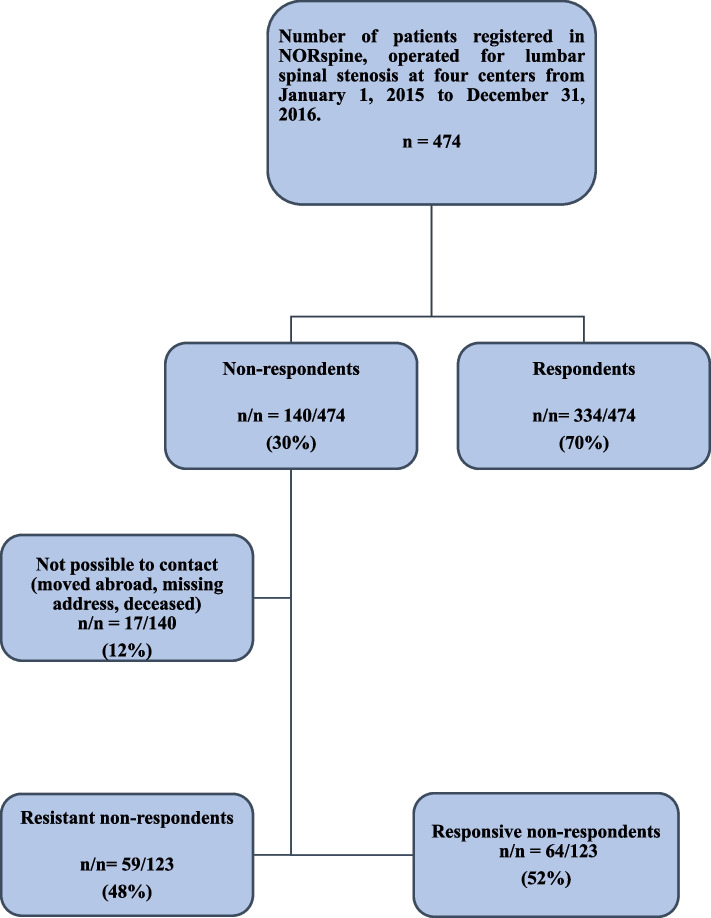
Study flowchart

At baseline, we compared 140 non-respondents with 334 respondents. In our cross-sectional analysis, we were able to contact 123 of the 140 non-respondents—17 were classified as “unknown address”, “moved abroad”, or “deceased” (Fig. 1). We then compared clinical outcomes assessed by PROMs between responsive non-respondents (median 50 months after surgery) and respondents (12 months after surgery). We also dichotomized clinical outcome using the GPE scale, defining success as “completely recovered” and “much improved”, and compared the proportions of successfully treated non-respondents versus respondents.

Finally, we compared the baseline characteristics of the responsive non-respondents and the resistant non-respondents.

### Statistics

We used descriptive statistics presented by means (SD) for continuous variables and numbers (percentages) for categorical variables. We analyzed between-group differences by mean difference (95%CI) and Student’s T-test for continuous variables, or relative risk (95%CI) and z-statistics for categorical variables. Statistical analyses were performed using SPSS, version 26 (IBM Corp., Armonk, N.Y. USA) and MedCalc Software Ltd. Relative risk calculator. https://www.medcalc.org/calc/relative_risk.php (Version 20.027; accessed March 14, 2022).

### Ethical considerations

All patients provided an informed consent when entering the registry. The Norwegian national ethical board (Regional Committee for medical and health research ethics, reference number 2017/2157) approved this study, as did the data protection officers at the four participating hospitals. This study was conducted in accordance with the Helsinki declaration [15].

## Results

As seen in Fig. 1, of the 474 consenting patients, 140 (30%) patients did not return the questionnaire at 12 months postoperatively. At the time of cross-sectional data collection, 17 patients were not possible to contact, leaving 123 for analysis. Of the 123 non-respondents, 64 (52%) patients returned questionnaires (“responsive non-respondents”), while 59 (48%) failed to respond (“resistant non-respondents”).

## Baseline characteristics

The non-respondents were younger than the respondents, 63 (SD 11.7) vs. 68 (SD 9.9) years, mean difference (95% CI) 4.7 years (2.59 to 6.74); *p* =  < 0.001. Non-respondents were more frequently smokers compared to respondents: 41 (30%) vs. 70 (21%), RR (95%CI) 1.40 (1.01 to 1.95); *p* = 0.044. Furthermore, non-respondents had a lower proportion of surgeon-reported relevant comorbidities compared to respondents 93 (69%) vs. 243 (78%), RR (95%CI) 0.89 (0.77 to 1.00); *p* = 0.047. However, we found no difference in ASA classification between non-respondents and respondents: the number (%) of ASA grades 1 and 2 was 111 (79%) vs. 242 (72%) RR (95% CI) 1.09 (0.98 to 1.22); *p* = 0.100. As shown in Table 1, there were no other differences between the non-respondents and respondents at baseline. Also, we found no differences in the type of surgery (decompression only vs. decompression and additional fusion) among the non-respondents and respondents.

**Table 1:** Baseline characteristics and perioperative data of 474 patients with lumbar stenosis who reported to NORspine

	**N** **Missing**	**Non-respondents (SD, %)**	**N** **Missing**	**Respondents (SD, %)**	**Mean difference (95% CI) or relative risk (95% CI)**	*P* value
**Age (years)**	N = 140 Missing = 0	63.0 (11.7)	N = 334 Missing = 0	67.7 (9.9)	-4.7 (-6.74 to -2.59)	< 0.001
**Female**	N = 140 Missing = 0	71 (51%)	N = 334 Missing = 0	183 (55%)	0.93 (0.77 to 1.12)	0.426
**BMI**	N = 136 Missing = 4	28.6 (4.5)	N = 329 Missing = 5	28.2 (4.5)	0.4 (-0.48 to 1.33)	0.362
Comorbidities*	N = 135 Missing = 5	93 (69%)	N = 310 Missing = 24	243 (78%)	0.89 (0.77 to 1.00)	0.047
**ASA grade I and II**	N = 140 Missing = 0	111 (79%)	N = 334 Missing = 0	242 (72%)	1.09 (0.98 to 1.22)	0.100
**Smokers**	N = 138 Missing = 2	41 (30%)	N = 331 Missing = 3	70 (21%)	1.40 (1.01 to 1.95)	0.044
**Norwegian as first language**	N = 138 Missing = 2	130 (94%)	N = 331 Missing = 3	324 (98%)	0.96 (0.92 to 1.01)	0.090
**University or college education > 4 years**	N = 136 Missing = 4	30 (22%)	N = 328 Missing = 6	83 (25%)	0.87 (0.60 to 1.26)	0.463
**Single civil status**	N = 139 Missing = 1	30 (22%)	N = 332 Missing = 2	85 (26%)	0.84 (0.58 to 1.22)	0.361
**Preoperative ODI**	N = 136 Missing = 4	42.3 (16.1)	N = 329 Missing = 5	40.4 (15.8)	1.87 (-1.31 to 5.06)	0.248
**Preoperative NRS back pain**	N = 127 Missing = 13	6.9 (2.0)	N = 312 Missing = 22	6.8 (2.1)	0.17 (-0.26 to 0.61)	0.430
**Preoperative NRS leg pain**	N = 123 Missing = 17	6.9 (2.2)	N = 311 Missing = 23	7.0 (2.1)	-0.04 (-0.49 to 0.40)	0.844
**Decompression only type surgery**	N = 140 Missing = 0	122 (87%)	N = 334 Missing = 0	301 (90%)	0.98 (0.84 to 1.15)	0.820
**Fusion type surgery**	N = 140 Missing = 0	18 (13%)	N = 334 Missing = 0	33 (10%)	1.27 (0.74 to 2.18)	0.393

^*^ Comorbidities that were assessed as relevant by the reporting surgeon

### Clinical outcomes

As presented in Table 2, we did not find any differences in mean (SD) ODI scores between the responsive non-respondents and respondents postoperatively 28.2 (19.9) vs. 25.2 (18.9), mean difference (95% CI) = 3.0 (-2.1 to 8.1); *p* = 0.250. Nor did we find any differences between responsive non-respondents versus respondents for NRS back pain, 4.6 (3.0) vs. 4.1 (2.9), mean difference (95% CI) 0.43 (-0.3 to 1.2); *p* = 0.271 or NRS leg pain score 4.0 (3.2) vs. 3.9 (3.1) mean difference (95% CI) 0.15 (-0.7 to 1.0); *p* = 0.719. Finally, we found similar proportions of successively treated patients among non-respondents and respondents, as assessed by GPE (63 (70%) vs. 330 (79%), RR (95%CI) 0.89 (0.75 to 1.06); *p* = 0.183).

**Table 2:** Postoperative clinical outcomes for responsive non-respondents and respondents operated for lumbar spinal stenosis

	**N** **Missing**	Responsive non-respondents* Mean (SD) / n (%)	**N** **Missing**	Respondents** Mean (SD)/ n (%)	**Mean diff (95% CI) or** **Relative risk (95% CI)**	*P*-value
ODI	N = 64 Missing = 0	28.2 (19.9)	N = 333 Missing = 1	25.2 (18.9)	2.99 (-2.1 to 8.1)	0.250
NRS back pain	N = 64 Missing = 0	4.6 (3.0)	N = 328 Missing = 5	4.1 (2.9)	0.43 (-0.3 to 1.2)	0.271
NRS leg pain	N = 63 Missing 1	4.0 (3.2)	N = 321 Missing = 12	3.9 (3.1)	0.15 (-0.7 to 1.0)	0.719
Success by GPE***	N = 63 Missing 1	63 (70%)	N = 330 Missing = 3	330 (79%)	0.89 (0.8 to 1.1)	0.183

^*^ PROM scores collected retrospectively at a median of 50 months after surgery

^**^PROM scores collected prospectively at 12 months after surgery

^***^Success defined as “completely recovered” or “much recovered” on the GPE scale

### Resistant non-respondents

Appendix Table 1 compares the responsive non-respondents (64 (52%)) to resistant non-respondents (59 (48%)). We did not find any age difference; however, resistant non-respondents were more frequently smokers (22 (38%) vs. 13 (20%), RR (95% CI) 1.87 (1.04 to 3.36); p = 0.037). As shown in Appendix Table 1, we did not find differences in other baseline characteristics such as sex, marital status, level of education, native language, ASA grade, or preoperative PROM levels.

## Discussion

The main findings from this register-based cohort study of patients who had spinal surgery due to lumbar spinal stenosis were that non-respondents were somewhat younger and tended to smoke more often than those who responded. Moreover, we found no differences in PROM scores between non-respondents compared to respondents, neither at baseline nor after surgery.

Several studies have demonstrated that non-respondents are younger than respondents [2, 4, 16–19]. Completing and posting questionnaires consumes time, and younger patients may be busier due to work and family obligations. Our finding that non-respondents were more frequently smokers has also been supported by others [2, 4, 6, 17, 18]. Also, we found that surgeons reported fewer relevant comorbidities for non-respondents than respondents. However, the variable “relevant comorbidity” is subject to interpretation by the treating surgeon. Therefore, the registration of relevant comorbidities by the treating surgeon may be questioned. A validation study of NORspine data found that surgeons tended to underreport relevant comorbidities and that ASA grading done by the anesthetist could be more reliable in assessing comorbidity [20]. In our study, there was no difference in the proportions of ASA grades 1 and 2 patients among non-respondents compared to respondents.

In addition to young age and smoking, previous studies of non-respondents also reported a predominance of the male gender, living alone, higher anxiety levels, and worse PROM scores [2, 4–6, 16–19]. Two observational spine studies found that non-respondents had higher ODI scores, lower quality of life (EuroQol 5D), and lower function (Short form health survey—SF-36) preoperatively compared to those who responded [2, 19]. The aforementioned studies implied that non-respondents had a worse starting point and were not quite representative of the entire register population. However, these findings were not reproduced in our study. Neither at baseline nor at follow-up did we find any differences in ODI between the non-respondents and respondents (Tables 1 and 2).

Another Swedish spine register study reported that non-respondents had inferior clinical outcomes [6], while other studies support our findings of similar postoperative outcomes for non-respondents versus respondents [2, 4, 5, 16–18]. Minor differences in PROMs have been reported between non-respondents and respondents, but the magnitudes of these differences were assessed as clinically irrelevant [21].

Some studies suggest that loss to follow-up of as little as 5% [22, 23] may cause bias, while rates above 20% [24] could potentially lead to serious bias. There is a variation in loss to follow-up rates in spine register studies ranging from 12% [4] to 42% [2]. The loss to follow-up at 12 months after surgery in our study was 30%. Moreover, previous studies have implied that it is not the extent of loss to follow-up but the type of attrition that is relevant for the assessment of bias [1, 7, 25]. Classification of missing data based on Rubin´s and Little´s work differentiates between data missing at random (MAR), missing completely at random (MCAR), and missing at non-random (MNAR) [26]. In cases of MAR, the non-respondents and respondents differ at baseline but report similar clinical outcomes after treatment; in cases of MCAR, the groups are similar at baseline and report similar outcomes; in cases of MNAR, the two groups compared report different outcomes. The largest risk of bias in a registry setting arises in cases of MNAR—the results are based on respondents only [1]. The use of multiple imputations and mixed linear models are used to manage MNAR [25]. Parai et al. found the loss to follow-up in the Swedish spine registry to be of the MNAR type [6], while Solberg et al. and Højmark et al. found MAR as the mechanism of loss to follow-up in the Norwegian and Danish spine registries [4, 5]. In our study, data seem to be missing at random since baseline characteristics differ somewhat between non-respondents and respondents, but the two groups report similar outcomes.

The methods used by registries to collect data may influence patient response. Reasons for patients not responding can be related to forgetfulness, lack of interest, and questionnaires being too time demanding. Clinical visits and telephone interviews have been shown to increase response rates [5], but they are time-consuming, costly, and not practical in a register setting. A web-based registry has shown a high loss to follow-up (59%) [17]. A combination of postal and web-based methods could complement each other and increase response rates. NORspine plans to implement a combination of methods to increase the follow-up rate.

### Strengths and limitations

The main weaknesses of our study are that we reached out to a sample of all potential register patients and that responsive non-respondents were compared to respondents at different time points, i.e., 12 months vs. 50 (36–64) months after surgery. However, previously published data have shown that patients who are followed longer than one year after spinal surgery keep reporting stable symptoms [27].

## Conclusion

In this observational study based on data from a national spine registry, we found a 30% loss to follow-up at 12 months after surgery for lumbar spinal stenosis. We reached out to non-respondents after surgery and found that non-respondents were somewhat younger and more frequently smokers. However, non-respondents reported similar clinical outcomes compared to those who responded. Our findings suggest that attrition bias in NORspine was random and due to non-modifiable factors.

## Supplementary Information

**Additional file 1.**

## Data Availability

The datasets generated and analysed during the current study are not publicly available due to the Norwegian data protection law but are available from the corresponding author at reasonable request.

## Abbreviations

NORspine: Norwegian registry for spine surgery
ODI: Oswestry Disability Index
NRS: Numerical rating scale
PROM: Patient-reported outcome measure
GPE: Global Perceived Effect
EQ-5D: European quality of life 5-dimension questionnaire
SF-36: Short form- 36 health survey
MAR: Missing at random
MNAR: Missing not at random
MCAR: Missing completely at random
