# Supplementary material for: Characteristics and outcomes of patients who did not respond to a national spine surgery registry
Source: BMC Musculoskelet Disord. 2023 Mar 4;24:164. doi: 10.1186/s12891-023-06267-3 (PMC9985292; doi:10.1186/s12891-023-06267-3)
Supplement: Supplementary file 1 — Additional file 1. [file 12891_2023_6267_MOESM1_ESM.docx]

| **Appendix Table 1  Baseline characteristics of responsive non-respondents (n=64) and resistant non-respondents (n=59).** | | | | |
| --- | --- | --- | --- | --- |
|  | **Responsive non-respondents mean (SD)/n (%)** | **Resistant non-respondents mean (SD)/n (%)** | **Mean difference (95% CI) or Relative risk (95% CI)** | **P-value** |
| **Age** | 64.6 (SD 10.2) | 63.2 (SD 11.1) | MD -1.5 (-5.28 to 2.32) | 0.445 |
| **Female** | 34 (53%) | 30 (51%) | RR 0.97 (0.65 to 1.45) | 0.887 |
| **Civil status – single** | 14 (22%) | 12 (20%) | RR 0.94 (0.47 to 1.90) | 0.867 |
| **University or college education > 4 years** | 7 (11%) | 6 (11%) | RR 1.07 (0.38 to 3.00) | 0.905 |
| **BMI** | 28.6 (SD 3.94) | 29.1 (SD 5.14) | MD 0.5 (-1.13 to 2.18) | 0.529 |
| **Smokers** | 13 (20%) | 22 (38%) | RR 1.87 (1.04 to 3.36) | 0.037 |
| **Norwegian as first language** | 53 (91%) | 60 (95%) | RR 0.96 (0.87 to 1.06) | 0.401 |
| **ASA grade I or II** | 53 (83%) | 42 (71%) | RR 1.09 (0.80 to 1.48) | 0.582 |
| **Comorbidities** | 45 (73%) | 39 (70%) | RR 1.02 (0.74 to 1.42) | 0.885 |
| **Preoperative ODI** | 41.9 (SD 16.20) | 44.3 (SD16.20) | MD 2.4 (-3.35 to 8.16) | 0.410 |
| **Preoperative NRS back** | 6.7 (SD 2.19) | 7.2 (SD 1.87) | MD 0.5 (-0.28 to 1.27) | 0.210 |
| **Preoperative NRS leg** | 7.0 (SD 2.08) | 7.1 (SD 2.17) | MD 0.1 (-0.69 to 0.94) | 0.762 |
